# Recent Molecular Advances in Our Understanding of Glioma

**DOI:** 10.7759/cureus.287

**Published:** 2015-07-23

**Authors:** Rohan Ramakrishna, David Pisapia

**Affiliations:** 1 Neurological Surgery, Weill Cornell Medical College; 2 Neurological Surgery, NewYork-Presbyterian/Weill Cornell Medical Center; 3 Pathology, Weill Cornell Medical College; 4 Pathology, New York Presbyterian Hospital

**Keywords:** neuro-oncology, glioma, glioblastoma, precision medicine, tert, 1p/19q, idh

## Abstract

Our molecular understanding of glioma has undergone a sea change over the last decade. In this review, we discuss two recent articles that employed whole genome sequencing to subclassify gliomas vis-à-vis known molecular alterations. We further discuss the relevance of these findings vis-à-vis current treatment paradigms.

## Introduction and background

Diffusely infiltrating gliomas are often persistent and aggressive lesions for which, despite decades of research, long-term control remains elusive. A major development in glioma biology recently pertains to our understanding of its molecular subgroups. These have included divisions into transcriptomal subtypes as well as analyses of glioma molecular evolution [1-5]. While gliomas are known for their genetic heterogeneity which relates to their treatment resistance, it is becoming increasingly apparent that gliomas do fall within distinct molecular subgroups that can generally predict outcomes. As of now, however, treatments based specifically on these molecular classifications have not become mainstream or standardized in the post-Stupp era [6].

## Review

Two studies recently published in the New England Journal of Medicine add to this body of knowledge [7-8]. In the first of these, Eckel-Passow, et al. hypothesized that stratification of gliomas based on alterations in the TERT promoter, *IDH *(including IDH1 and IDH2 mutations), and co-deletion of *1p19q *would identify groups with similar clinical variables, acquired somatic alterations, and germline variants. These alterations were selected for study given their prevalence within glioma, their presence as early alterations in the molecular evolution of glioma, and their strong association with overall survival based on previous clinical studies. Specifically, *TERT* encodes telomerase which is essential for telomere maintenance (shortened telomeres impede cellular division) and mutations in its promoter are often found in both oligodendroglioma and glioblastoma. As such, telomere maintenance emerges as a common molecular theme across markedly distinct subtypes of diffusely infiltrating glioma. As an aside, *TERT* is also interesting from the standpoint of aging (telomerase activity usually declines with aging), as one hallmark of glioma is worse prognosis of elderly patients compared to younger patients irrespective of co-morbid conditions [9]. Additionally, evidence has accumulated that the age of glioma stem cells contributes to their overall malignancy, perhaps due to the differing genomic landscape of the aged stem cell versus the younger one [10]. Another mutation investigated in this study was the *IDH *mutation, which is associated with the accumulation of a metabolite 2-hydroxyglutarate and also associated with improved prognosis [11]. Finally, co-deletion of 1p and 19q was assessed, given its association with chemotherapeutic response and the oligodendroglioma phenotype [12-14].

In this first study, 1,087 gliomas were analyzed and stratified into five groups based on these molecular characteristics and are presented in Tables 1-2. These cases included 317 cases from an initial discovery set and an additional 770 cases over two replication sets, including cases from the Cancer Genome Atlas.

**Table 1:** Molecular strata of 1,087 gliomas Adapted from Eckel-Passow, et al. [8].

Grade II/III Gliomas	Prevalence
Triple positive (IDH+, TERT mutation, 1p19q codeleted)	29%
IDH+ and TERT	5%
IDH+	45%
Triple negative (IDH-, TERT -, 1p19q intact)	7%
TERT+	10%
Other	5%
Grade IV Glioblastoma	
Triple positive (IDH+, TERT mutation, 1p19q codeleted)	1%
IDH+ and TERT	2%
IDH+	7%
Triple negative (IDH-, TERT -, 1p19q intact)	17%
TERT+	74%

**Table 2:** Molecular features of 1,087 gliomas. Adapted from Eckel-Passow, et al. [8].

Subtype	Features
Triple Positive	CIC/FUBP1/NOTCH1/PIK3CA/PIK3R1 mutations Loss of chromosome 4, hemizygous loss of CDKN2A/B Proneural GBM transcriptomal subtype
TERT and IDH mutations	TP53 and ATRX mutations Gain of chromosome 7, MYC duplication, deletion of PTEN, homozygous loss of CDKN2A/B Mesenchymal/neural/pro-neural GBM transcriptomal subtypes
IDH mutation	TP53 and ATRX mutations Duplication of 7q, MYC duplication, hemizygous loss of CDKN2A/B, deletion of 19q Proneural transcriptomal subtype
Triple negative	Loss of chromosome 4, gain of chromosome 7, gain of chromosome 19, amplification of EGFR, homozygous loss of CDKN2A/B, deletion of PTEN, other amplifications
TERT mutation	Loss of chromosome 4, gain of chromosome 7, gain of chromosome 19, amplification of EGFR, homozygous loss of CDKN2A/B, deletion of PTEN, other amplifications Classical/Mesenchymal GBM transcriptomal subtypes

An interesting takeaway from this data concerns its concordance with what is known about primary and secondary glioblastoma, and age-related features in glioblastoma. For example, in this study, standalone IDH mutations were significantly more frequent in younger patients and seemed to go along with tumor evolution along a secondary glioblastoma pathway. Similarly, patients whose tumors harbored *TERT* mutations tended to be much older and their tumors also frequently showed EGFR alterations, again more consistent with elderly populations harboring primary glioblastoma. Finally, survival analysis revealed that patients (adjusted for age and grade) harboring *TERT* mutations suffered worse overall survival compared with the other molecular subgroups. Similarly, patients with triple negative gliomas had poorer overall survival than gliomas with *TERT*, *IDH,* or triple positive gliomas.  Of note among Grade IV gliomas, the molecular subgroups assigned in this study were not associated with survival differences in multivariate analyses.

**Figure 1:**
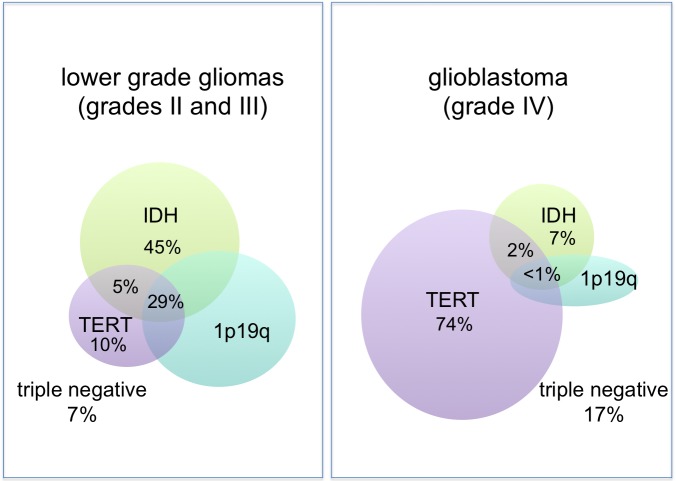
Molecular strata of 1,087 gliomas Adapted from Eckel-Passow, et al. [8].

This study was important as it found consistent associations between their a priori identified molecular groups and age at diagnosis, survival, patterns of acquired alterations, and germline variants. It also confirmed the importance of the *IDH* mutation as an important biologic target. Moreover, it showed that *IDH* mutations are not always favorable. In particular, among patients with a histopathologic diagnosis of glioblastoma, patients with both* TERT *and *IDH* mutations had poor overall survival, similar to patients with *TERT* mutations only. The study also demonstrated a relationship between *TERT* mutations and germline variants in telomere components (TERC/*TERT*/RTEL1), which is relevant given interest in telomeres and cancer more generally [15]. Similarly, it was confirmed that SNPs at chromosome locus 8q24 were highly associated with the *IDH* mutation, which suggests that this region contains a germline alteration that facilitates the development of *IDH* mutant gliomas.  

In a companion study published in the same issue of the June 2015 New England Journal of Medicine, the TCGA Research Network published a whole genome analysis of 293 adult lower grade gliomas and correlated this data with clinical outcomes. In short, their study demonstrated that clinical outcome was better predicted by molecular subclasses dictated by *IDH*, *1p19q*, and TP53 status than by traditional histopathologic diagnosis. Similar to the previous study, the TCGA study found that patients with *IDH* mutations and *1p19q* co-deletions had the most favorable prognosis and a strong histologic correlation with oligodendroglioma. Moreover, this class of patients frequently harbored mutations in CIC, FUBP1, NOTCH1, and the *TERT* promoter. In contrast, those gliomas with *IDH* mutations but lacking *1p19q* co-deletion had mutations in TP53 as well as ATRX inactivation and were generally associated with astrocytic histomorphology, including those tumors with mixed morphologies. The propensity to achieve gross total resection did not differ by molecular class. Finally, those lower grade tumors without *IDH* mutations had clinical behavior highly similar to glioblastoma.

Interestingly, the authors queried the genomic data from these gliomas and were able to find clusters within groups related to DNA methylation, gene expression, DNA copy number, and microRNA expression. They then integrated this data with the molecular strata data and histologic subtypes to generate a cluster of cluster analysis. Consequently, they were able to show that classifying tumors based on *IDH* and *1p19q* status mapped universally to a specific cluster, whereas histologic designation (i.e., oligodendroglioma, astrocytoma, and oligoastrocytoma) matched one-to-one with a cluster only 63% of the time. This underscores the inability of morphological and other often subjective histological criteria to reflect that broader molecular profile of a particular tumor and suggests that molecular markers are a more reliable way to define clinically relevant diagnostic entities that would be more reflective of their biologic potential. An additional finding was that the background mutational frequency of *IDH* wild-type tumors was significantly elevated compared to *IDH* mutant tumors. This was repeated and validated with another genomic analysis approach (OncoSign) that confirmed these findings.

In lower grade gliomas with *IDH* mutations and *1p19q* co-deletion, the authors found frequent mutations in CIC, FUBP1, PI3 kinase pathway genes, NOTCH1, ZBTB20, and ARIDIA, in addition to activating *TERT* promoter mutations. Overall, the data suggested that lower grade gliomas with *IDH* mutations and *1p19q* co-deletions are biologically distinct and arise from a sequence of *IDH* mutation and *1p19q* deletion, *TERT*/PI3 kinase activation, and NOTCH1 inactivation. In IDH mutant tumors without *1p19q* deletion, TP53 mutations were most frequent along with inactivating mutations in ATRX. *TERT* mutations were rare, but mutations in ATRX are associated with the alternative mechanism of lengthening telomeres (ALT) process.

In addition, protein pathway analysis revealed and highlighted the similarities between *IDH* wild-type lower grade tumors and glioblastoma. For example, activation of receptor kinase pathways (i.e., EGFR) was manifold more frequent in *IDH* wild-type tumors compared to *IDH* mutants. In addition, *IDH* wild-type tumors over-expressed HER2. In general, however, these protein expression profiles highlighted the fundamental background biologic difference between *IDH* mutant and *IDH* wild-type tumors.

### Upshot

The two studies are notable in that they genomically validate the utility of previously reported molecular markers; for example, that *IDH* status and *1p19q* co-deletion are more important prognostically than standard histopathologic diagnosis. This is exemplified by the fact that *IDH* wild-type infiltrative astrocytomas with a lower histologic grade have a similar prognosis to that of (Grade IV) GBM.  Additionally, these studies confirm that lower-grade gliomas with an *IDH* mutation have either *1p/19q* co-deletion or a TP53 mutation, with few gaps or overlaps, reflecting two distinct molecular mechanisms of oncogenesis. This finding supports eliminating the designation “oligoastrocytoma”, a diagnostic entity of notoriously high inter-observer variability that is often a source of confusion. 

Moreover, these studies underscore that the biology of gliomas, when stratified by molecular subgroup, can differ substantially in terms of their molecular evolution, mutational landscape, and clinical behavior. Indeed, the elegance of these studies rests in their power to meaningfully classify gliomas based on a small set of markers that can be queried routinely in the clinic in a way that captures the broader underlying genomic landscape of a tumor, as previous transcriptomal studies attempted to do [1-2]. It is also worth noting that these studies confirmed that age plays an important independent prognostic role, particularly in the Eckel-Passow study that mirrored previous clinical papers that prognosticated glioma [16-17].

These studies also raise important questions and concerns regarding future treatments. For example, what are the practical considerations of developing trials and accruing patients with lower grade gliomas based on molecular parameters? Given the TCGA data especially, should low grade *IDH *wild-type tumors be treated like glioblastoma initially with adjuvant chemoradiation? Also, does initial surgery/reoperation or “supratotal” resection change the natural history of this disease, and is this response dependent on the molecular strata [18-19]? Finally, while the sampling error in histopathologic diagnosis is a well-recognized problem, can sampling error also lead to misclassification of gliomas on a molecular level, given the genomic heterogeneity of, for example, GBM, and other tumor types [20]?

Other questions abound. For example, why do patients with Grade II/III tumors in the Eckel-Passow study with *TERT* mutations and *TERT* and *IDH* mutations differ wildly in terms of survival while they co-register fairly well among Grade IV tumors? As alluded to in the study, among lower grade tumors, it is possible that a subset of the *TERT/IDH* double positives also contain alterations with functional equivalence to that of *1p/19q* deletion, without this latter alteration per se. On the other hand, among Grade IV tumors, since there were only 11 *TERT/IDH* mutant tumors in the Grade IV group, compared to 347 in the *TERT*-only group, it is possible that this comparison lacked sufficient power to detect a survival advantage. Certainly, this question is worth pursuing. Also, as alluded to by the authors, an open question remains the clinical impact of the cancer methylome in light of recent data, including that of dynamic methylation patterns (TET proteins) in cancer [21-22].

The final question, of course, is the extent to which these analyses reveal clinically relevant distinct entities among diffusely infiltrating gliomas, including GBM. Apart from *IDH* mutant tumors, the survival curves of glioblastoma in the Eckel-Passow study are very similar, with *IDH* wild-type tumors performing poorly (particularly the *TERT* mutant subset). As such, are these molecular strata purely academic or will treatments be devised that take advantage of these molecular differences? Certainly, analysis of long-term survivors based on transcriptomal patterns did not show a preponderance of survivors in one transcriptomal subgroup versus another in previous studies [23]. It should be emphasized that as molecular markers are increasingly incorporated as diagnostic criteria, the essential principles that dictate the utility of a ‘diagnostic entity’ should be retained; that is, a diagnosis is useful only when it guides treatment decisions and offers prognostic information as specifically as possible. The development of diagnostic schemata is an iterative process with inputs including an evolving knowledge of tumor biology and clinical outcomes. These studies have provided important information that more rigorously classifies these tumors, with hopes that this improved understanding will lead to improved clinical care.

## Conclusions

In this review, we have discussed the importance of two recent studies utilizing whole exome sequencing to subclassify gliomas. The clinical utility in terms of decision making is yet to become standard, but these studies are an important step towards understanding the fundamental biologic mechanisms that govern gliomas.

## References

[1] Phillips HS, Kharbanda S, Chen R, Forrest WF, Soriano RH, Wu TD, Misra A, Nigro JM, Colman H, Soroceanu L, Williams PM, Modrusan Z, Feuerstein BG, Aldape K (2006). Molecular subclasses of high-grade glioma predict prognosis, delineate a pattern of disease progression, and resemble stages in neurogenesis. Cancer Cell.

[2] Verhaak RG, Hoadley KA, Purdom E, Wang V, Qi Y, Wilkerson MD, Miller CR, Ding L, Golub T, Mesirov JP, Alexe G, Lawrence M, O'Kelly M, Tamayo P, Weir BA, Gabriel S, Winckler W, Gupta S, Jakkula L, Feiler HS, Hodgson JG, James CD, Sarkaria JN, Brennan C, Kahn A, Spellman PT, Wilson RK, Speed TP, Gray JW, Meyerson M, Getz G, Perou CM, Hayes DN, Cancer Genome Atlas Research Network (2010). Integrated genomic analysis identifies clinically relevant subtypes of glioblastoma characterized by abnormalities in PDGFRA, IDH1, EGFR, and NF1. Cancer Cell.

[3] Johnson BE, Mazor T, Hong C, Barnes M, Aihara K, McLean CY, Fouse SD, Yamamoto S, Ueda H, Tatsuno K, Asthana S, Jalbert LE, Nelson SJ, Bollen AW, Gustafson WC, Charron E, Weiss WA, Smirnov IV, Song JS, Olshen AB, Cha S, Zhao Y, Moore RA, Mungall AJ, Jones SJ, Hirst M, Marra MA, Saito N, Aburatani H, Mukasa A, Berger MS, Chang SM, Taylor BS, Costello JF (2014). Mutational analysis reveals the origin and therapy-driven evolution of recurrent glioma. Science.

[4] Li Y, Wang D, Wang L (2013). Distinct genomic aberrations between low-grade and high-grade gliomas of Chinese patients. PLoS One.

[5] Marko NF, Prayson RA, Barnett GH, Weil RJ (2010). Integrated molecular analysis suggests a three-class model for low-grade gliomas: a proof-of-concept study. Genomics.

[6] Hegi ME, Diserens AC, Gorlia T, Hamou MF, de Tribolet N, Weller M, Kros JM, Hainfellner JA, Mason W, Mariani L, Bromberg JE, Hau P, Mirimanoff RO, Cairncross JG, Janzer RC, Stupp R (2005). MGMT gene silencing and benefit from temozolomide in glioblastoma. NEJM.

[7] Cancer Genome Atlas Research Network (2015). Comprehensive, integrative genomic analysis of diffuse lower-grade gliomas. NEJM.

[8] Eckel-Passow JE, Lachance DH, Molinaro AM, Walsh KM, Decker PA, Sicotte H, Pekmezci M, Rice T, Kosel ML, Smirnov IV, Sarkar G, Caron AA, Kollmeyer TM, Praska CE, Chada AR, Halder C, Hansen HM, McCoy LS, Bracci PM, Marshall R, Zheng S, Reis GF, Pico AR, O'Neill BP, Buckner JC, Giannini C, Huse JT, Perry A, Tihan T, Berger MS, Chang SM, Prados MD, Wiemels J, Wiencke JK, Wrensch MR, Jenkins RB (2015). Glioma groups based on 1p/19q, IDH, and TERT promoter mutations in tumors. NEJM.

[9] Kita D, Ciernik IF, Vaccarella S, Franceschi S, Kleihues P, Lütolf UM, Ohgaki H (2009). Age as a predictive factor in glioblastomas: population-based study. Neuroepidemiology.

[10] Mikheev AM, Ramakrishna R, Stoll EA, Mikheeva SA, Beyer RP, Plotnik DA, Schwartz JL, Rockhill JK, Silber JR, Born DE, Kosai Y, Horner PJ, Rostomily RC (2012). Increased age of transformed mouse neural progenitor/stem cells recapitulates age-dependent clinical features of human glioma malignancy. Aging Cell.

[11] Cairncross JG, Wang M, Jenkins RB, Shaw EG, Giannini C, Brachman DG, Buckner JC, Fink KL, Souhami L, Laperriere NJ, Huse JT, Mehta MP, Curran WJ Jr (2014). Benefit from procarbazine, lomustine, and vincristine in oligodendroglial tumors is associated with mutation of IDH. J Clin Oncol.

[12] Cairncross G, Wang M, Shaw E, Jenkins R, Brachman D, Buckner J, Fink K, Souhami L, Laperriere N, Curran W, Mehta M (2013). Phase III trial of chemoradiotherapy for anaplastic oligodendroglioma: long-term results of RTOG 9402. J Clin Oncol.

[13] Jenkins RB, Blair H, Ballman KV, Giannini C, Arusell RM, Law M, Flynn H, Passe S, Felten S, Brown PD, Shaw EG, Buckner JC (2006). A t(1;19)(q10;p10) mediates the combined deletions of 1p and 19q and predicts a better prognosis of patients with oligodendroglioma. Cancer Res.

[14] van den Bent MJ1, Brandes AA, Taphoorn MJ, Kros JM, Kouwenhoven MC, Delattre JY, Bernsen HJ, Frenay M, Tijssen CC, Grisold W, Sipos L, Enting RH, French PJ, Dinjens WN, Vecht CJ, Allgeier A, Lacombe D, Gorlia T, Hoang-Xuan K (2013). Adjuvant procarbazine, lomustine, and vincristine chemotherapy in newly diagnosed anaplastic oligodendroglioma: long-term follow-up of EORTC brain tumor group study 26951. J Clin Oncol.

[15] Walsh KM, Wiencke JK, Lachance DH, Wiemels JL, Molinaro AM, Eckel-Passow JE, Jenkins RB, Wrensch MR (2015). Telomere maintenance and the etiology of adult glioma. Neuro Oncol.

[16] Pignatti F, van den Bent M, Curran D, Debruyne C, Sylvester R, Therasse P, Afra D, Cornu P, Bolla M, Vecht C, Karim AB, European Organization for Research and Treatment of Cancer Brain Tumor Cooperative Group, European Organization for Research and Treatment of Cancer Radiotherapy Cooperative Group (2002). Prognostic factors for survival in adult patients with cerebral low-grade glioma. J Clin Oncol.

[17] Bauman G, Lote K, Larson D, Stalpers L, Leighton C, Fisher B, Wara W, MacDonald D, Stitt L, Cairncross JG (1999). Pretreatment factors predict overall survival for patients with low-grade glioma: a recursive partitioning analysis. Int J Radiat Oncol Biol Phys.

[18] Ramakrishna R, Hebb A, Barber J, Rostomily R, Silbergeld D (2015). Outcomes in reoperated low-grade gliomas. Neurosurg.

[19] Yordanova YN, Moritz-Gasser S, Duffau H (2011). Awake surgery for WHO Grade II gliomas within "noneloquent" areas in the left dominant hemisphere: toward a "supratotal" resection. Clinical article. J Neurosurg.

[20] Aubry M, de Tayrac M, Etcheverry A, Clavreul A, Saikali S, Menei P, Mosser J (2015). From the core to beyond the margin: a genomic picture of glioblastoma intratumor heterogeneity. Oncotarget.

[21] Huang Y, Rao A (2014). Connections between TET proteins and aberrant DNA modification in cancer. Trends Genet.

[22] Bian EB, Zong G, Xie YS, Meng XM, Huang C, Li J, Zhao B (2014). TET family proteins: new players in gliomas. J Neurooncol.

[23] Gerber NK, Goenka A, Turcan S, Reyngold M, Makarov V, Kannan K, Beal K, Omuro A, Yamada Y, Gutin P, Brennan CW, Huse JT, Chan TA (2014). Transcriptional diversity of long-term glioblastoma survivors. Neuro Oncol.

